# Effects of a Singular Dose of Mangiferin–Quercetin Supplementation on Basketball Performance: A Double-Blind Crossover Study of High-Level Male Players

**DOI:** 10.3390/nu16010170

**Published:** 2024-01-04

**Authors:** Dimitrios I. Bourdas, Antonios K. Travlos, Athanasios Souglis, Georgia Stavropoulou, Emmanouil Zacharakis, Dimitrios C. Gofas, Panteleimon Bakirtzoglou

**Affiliations:** 1 Section of Sport Medicine & Biology of Exercise, School of Physical Education and Sports Science, National and Kapodistrian University of Athens, 41 Ethnikis Antistasis, 17237 Daphne, Greece; dbourdas@phed.uoa.gr (D.I.B.); asouglis@phed.uoa.gr (A.S.); emzach@phed.uoa.gr (E.Z.); 2 Department of Sports Organization and Management, Faculty of Human Movement and Quality of Life Sciences, University of Peloponnese, Efstathiou and Stamatikis Valioti & Plataion Avenue, 23100 Sparta, Greece; atravlos@uop.gr; 3 School of Philosophy and Education, Aristotle University of Thessaloniki, University Campus, 54124 Thessaloniki, Greece; gnstavro@edlit.auth.gr; 4 Arsakeia-Tositseia Schools, Philekpaideftiki Etaireia, Mitilinis 26, 11256 Athens, Greece; gofasd@hotmail.com; 5 Faculty of Sport Sciences & Physical Education, Metropolitan College, Eleftheriou Venizelou 14, 54624 Thessaloniki, Greece

**Keywords:** antioxidant supplementation, ergogenic aids, fatigue, high-intensity exercise, sprint exercise, human subjects, metabolism, muscle function, performance, polyphenols

## Abstract

Pre-exercise mangiferin–quercetin may enhance athletic performance. This study investigated the effect of mangiferin–quercetin supplementation on high-level male basketball players during a basketball exercise simulation test (BEST) comprising 24 circuits of 30 s activities with various movement distances. The participants were divided into two groups (EXP = 19 and CON = 19) and given a placebo one hour before the BEST (PRE-condition). The following week, the EXP group received mangiferin–quercetin (84 mg/140 mg), while the CON group received a placebo (POST-condition) before the BEST in a double-blind, cross-over design. The mean heart rate (HR) and circuit and sprint times (CT and ST) during the BEST were measured, along with the capillary blood lactate levels (La^−^), the subjective rating of muscle soreness (RPMS), and the perceived exertion (RPE) during a resting state prior to and following the BEST. The results showed significant interactions for the mean CT (*p* = 0.013) and RPE (*p* = 0.004); a marginal interaction for La^−^ (*p* = 0.054); and non-significant interactions for the mean HR, mean ST, and RPMS. Moreover, the EXP group had significantly lower values in the POST condition for the mean CT (18.17 ± 2.08 s) and RPE (12.42 ± 1.02) compared to the PRE condition (20.33 ± 1.96 s and 13.47 ± 1.22, respectively) and the POST condition of the CON group (20.31 ± 2.10 s and 13.32 ± 1.16, respectively) (*p* < 0.05). These findings highlight the potential of pre-game mangiferin–quercetin supplementation to enhance intermittent high-intensity efforts in sports such as basketball.

## 1. Introduction

Modern basketball necessitates the possession of exceptional technical skills [1,2] and the ability to execute medium-to-high-intensity actions, lasting up to 15 s, and explosive muscle efforts of high-to-maximal intensity lasting 2–5 s [1,2,3]. These actions occur in a random sequence with varying recovery intervals, highlighting the multifaceted physical demands of the sport [4,5]. Despite the primary contribution of phosphocreatine and the fast glycolytic system to high-to-maximal-intensity sprint actions, the prolonged duration of the game and an average heart rate of 85% of the maximal heart rate (HR) during a match [3] underscore the significance of aerobic metabolism [2,4]. In essence, basketball is a physically demanding sport where success hinges on the mastery of fundamental physical characteristics, including acceleration, agility, strength, and power [1,2,3]. Furthermore, it is worth noting that psychological and nutritional factors can exert an influence on basketball performance [6,7]. Concerning the nutritional aspect, a plethora of functional foods and nutritional supplements have demonstrated ergogenic effects across various sports. However, many of these substances remain unexplored in the context of basketball, especially within field-based exercise testing protocols [7]. To our knowledge, the combination of mangiferin and quercetin supplements is one such uncharted territory [7].

Recently, research demonstrated the significant ergogenic potential of a combination of *Mangifera indica* L. leaf extract (MLE), which is rich in mangiferin, with quercetin [8]. This combination appears to increase muscle power in fatigued individuals, enhance peak oxygen uptake ($\dot{V}$O_2_), and improve brain oxygenation during extended sprinting efforts in females [8]. Mangiferin, a xanthone and non-flavonoid polyphenol, is notably abundant in mango leaves, bark, flowers, pulp, and various other plants [9] and exhibits a notable capacity for iron chelation along with potent antioxidant attributes [10]. Quercetin is a flavonoid polyphenol (occurring naturally in a variety of fruits and vegetables, including mangoes) with potential performance-enhancing properties during prolonged exercise [11,12,13] and has robust antioxidant and anti-inflammatory capabilities [14].

Natural polyphenols and antioxidants may modulate afferent signals emanating from group III and IV ergoreceptors [15,16], potentially enhancing muscle activation [13,17]. Furthermore, the combination of mangiferin and quercetin, when ingested within 48 h preceding repeated sprint exercises, can counteract the typical decline in brain oxygenation observed during prolonged sprinting [18] and enhance muscle oxygen extraction [8,19]. Oral administration of 500 mg of MLE has been linked to improved reaction time and reduced fatigue [20]. A single dose of 84 mg of mangiferin combined with 140 mg of quercetin, administered one hour prior to competition, followed by three additional doses every eight hours, has also shown promise in attenuating muscle pain, reducing damage, and expediting the recovery of muscle performance [21]. Thus, it seems that the synergistic effects of mangiferin and quercetin can mitigate fatigue through various molecular mechanisms [8,19]. However, these mechanisms remain unknown. Moreover, while the effects of a single dose of 84 mg of mangiferin combined with 140 mg of quercetin have been investigated in laboratory-based exercise protocols within the general population [22], their potential impact in field-based exercise protocols, especially among athletes participating in stop-and-go team sports such as basketball, remains largely unexplored.

In summary, there is a substantial body of evidence supporting the beneficial impact of mangiferin and quercetin supplement combinations on exercise performance among non-athlete populations [8,19,22]. However, there is a notable research gap when it comes to investigating the effects of this supplementation among athletes, particularly within the cohort of basketball players. Consequently, our primary objective was to assess the potential influence of mangiferin and quercetin supplementation on the performance of basketball players. We posited that this supplementation regimen would yield improvements in basketball performance. It is important to emphasize that this article predominantly delves into the scientific underpinnings of the potential ergogenic effects of combined mangiferin and quercetin supplementation on basketball performance rather than delving into the underlying physiological mechanisms responsible for these effects.

## 2. Materials and Methods

### 2.1. Participants

In this investigation, our focus was on a cohort of highly trained/national-level (tier 3 [23]) male basketball players (n = 38) representing the upper echelons of Greek basketball drawn from the top three national leagues. This cohort was stratified into two distinct groups: the experimental (EXP, Ne = 19) and control (CON, Nc = 19) groups. The groups were equally matched for the participants’ playing positions (EXP: seven guards, six forwards, and six centers; CON: six guards, seven forwards, and six centers) utilizing a randomized list in Excel. Detailed anthropometric and physiological characteristics of both groups are provided in Table 1. The selection criteria for participant inclusion encompassed individuals who had a minimum of six years of basketball-playing experience, were aged over 18 years, were highly trained/national-level basketball players (according to the McKay et al. criteria [23] and participants’ anthropometric and physiological traits [2,3,4,5]), and maintained a certain level of physical activity (i.e., at least moderate-to-vigorous physical activity, which entails a minimum of 60 min per day [24]) during the study period.

Conversely, exclusion criteria were applied to individuals who reported the use of mangiferin or quercetin within the preceding two weeks; had a documented history of clinically significant allergies or a known intolerance, hypersensitivity, or allergy to herbal extracts, mangiferin, or quercetin; had a history of smoking or the use of nicotine-related products; had musculoskeletal injuries occurring at least six months prior to the study; had significant respiratory, cardiovascular, or other severe medical conditions; had any ongoing medication regimen; or had a consistent daily sleep duration of less than 8 h. The participants’ self-reported levels of physical activity and well-being were assessed through the administration of the Active-Q and PAR-Q+ questionnaires [25,26], respectively. Additionally, the participants provided information on their smoking habits and perceived sleep adequacy [27].

**Figure 1:**
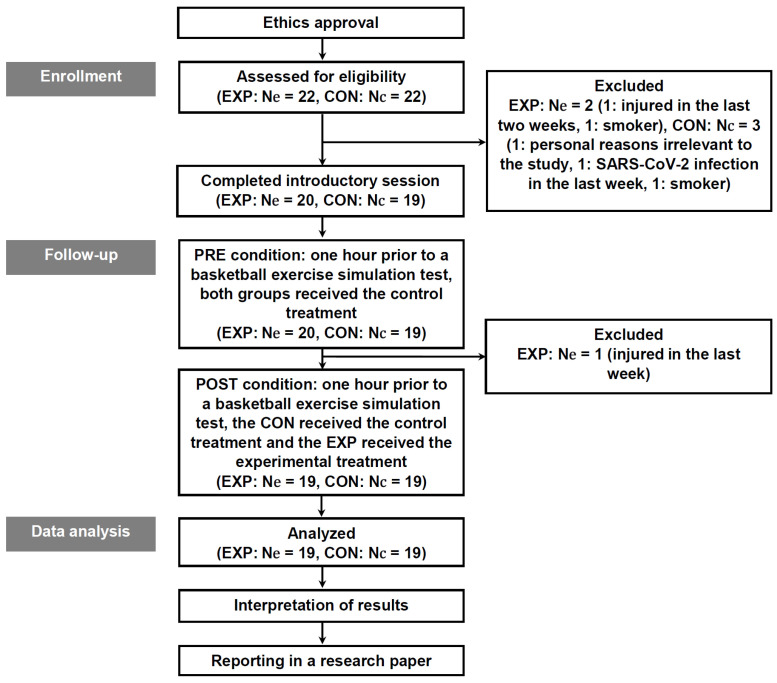
Experimental design. Abbreviations: CON, control group; EXP, experimental group; Nc, sample size of the control group; and Ne, sample size of the experimental group.

Ethical approval for this study was granted by the local University Committee on Human Research (protocol number: 3258/11 August 2023), and written informed consent was obtained from all participants. Each participant received comprehensive information regarding the study’s laboratory and field conditions and the employed methodologies, as well as potential risks, all in compliance with the most recent iterations of the ethical guidelines outlined in the Helsinki Declaration [28]. A graphical representation of the research design is illustrated in Figure 1.

### 2.2. Preliminary Measurements

At the outset of the study, during the initial laboratory visit, all participants were given a comprehensive orientation of the laboratory and court–field facilities and were provided with a detailed introduction to the specific methodologies that would be employed in this investigation. Next, measurements of physical characteristics were conducted with precision instruments: standing height was measured to the nearest 0.5 cm (Stadiometer; Seca, Birmingham, UK), and nude body mass was recorded to the nearest 0.1 kg (Beam Balance 710; Seca, Birmingham, UK). Moreover, the thicknesses of seven (chest, axilla, triceps, subscapula, abdomen, supra-iliac, and thigh) skinfolds [29] were determined using Harpenden Skinfold Calipers (Baty International, West Sussex, UK). Subsequently, body fat (BF) was estimated using established equations specific to these skinfold measurements and the study population [30].

The assessment of lower limb power in the participants involved the execution of a counter-movement jump (CMJ) that included an arm swing component on a contact platform (EuroJump^®^, Newtest, Oulu, Finland). Next, the maximal oxygen uptake ($\dot{V}$O_2_max) was assessed utilizing a metabolic cart (Vacumed Mini-CPX, Ventura CA, USA), which underwent prior calibration using known oxygen and carbon dioxide gas mixtures. The evaluation was conducted during an incremental treadmill (Technogym Runrace; Technogym, Gambettola, Italy) exercise test to exhaustion. The $\dot{V}$O_2_max protocol involved an initial running speed of 7 km·h^−1^ for 1 min, followed by an increase to 8 km·h^−1^ for 30 s. Subsequently, the treadmill speed was augmented by 0.5 km·h^−1^ every 30 s until the point of exhaustion was reached. Throughout the $\dot{V}$O_2_max test, the treadmill maintained a 1% incline. The $\dot{V}$O_2_max and maximum heart rate (HRmax) were identified as the highest values achieved within 15 s and 5 s, respectively, during the final phase of the incremental exercise. The attainment of $\dot{V}$O_2_max adhered to specific criteria, including the presence of at least two of the following: a respiratory exchange ratio exceeding 1.1, HRmax falling within 10 beats per minute of the estimated HRmax based on age, a rating of perceived exertion equal to or greater than 18, or a plateauing of $\dot{V}$O_2_max (<2 mL·kg^−1^·min^−1^) accompanied by an associated increase in treadmill speed [31]. Heart rate was measured telemetrically (Polar RCX5, Polar Electro Oy, Kempele, Finland), and the subjective rating of perceived exertion (RPE) was estimated using the 6–20 linear Borg scale [32].

**Figure 2:**
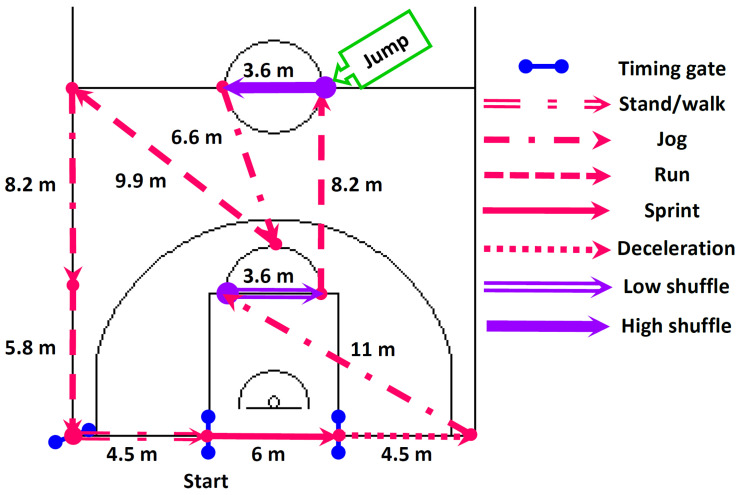
The basketball exercise simulation test (BEST) comprising 24 circuits of 30 s activities involving varying movement distances [33]. Standing or walking: activity at an intensity no greater than a brisk walking pace; jogging: activity at a moderate intensity level, exceeding walking pace but without a sense of urgency, at approximately 50% of maximal velocity; running: activity at an intensity higher than moderate, characterized by effort and purpose but not reaching maximal exertion, at approximately 75% of maximal velocity; sprinting: high-intensity, all-out effort performed at maximal velocity; low-intensity shuffling: activity featuring a shuffling foot movement within a defensive stance executed without a sense of urgency; high-intensity shuffling: activity involving a shuffling foot movement within a defensive stance performed at maximal effort; maximal-effort jumping: initiating a countermovement jump with maximal effort, pushing off with both legs. Participants aimed to complete a maximum of 24 circuits within the 12 min test duration. If a circuit could not be completed in 30 s, no rest was given, and participants immediately started the next circuit. In such cases, the targeted 24 circuits were not achieved unless normal timing was restored.

Following a recovery period of approximately 30 min after the $\dot{V}$O_2_max test, the participants underwent additional familiarization with the basketball exercise simulation test (BEST) protocol [33], which is depicted in Figure 2. This familiarization process entailed participants engaging in practice runs of the BEST at varying speeds, followed by the completion of approximately 6–8 circuits at full intensity [33].

### 2.3. Experimental Procedures

Seven days later, during the second visit, the participants performed the BEST on a hardwood indoor basketball court with official dimensions (PRE condition). One week later, the participants repeated the aforementioned protocol (POST condition). The participants were advised to consistently execute the test with maximal effort in their accustomed manner whenever it was performed, including when they had pre-consumed a capsule, which was either an experimental or placebo supplement.

The participants were extensively informed about the necessity of maintaining consistent sleeping patterns, dietary habits, and physical activity levels leading up to all successive trials. Specifically, in the week leading up to the second visit, participants were provided with specific dietary guidelines that emphasized the consumption of a well-balanced diet. This balanced diet was defined as one comprising approximately 50–60% of total energy intake from carbohydrates, 25–30% from fats, and 15% from proteins. The participants were also instructed to maintain meticulous dietary records, documenting both the ingredients and portion sizes of their meals in as much detail as possible. Furthermore, the participants were encouraged to replicate their previously recorded dietary patterns to the best of their ability during the subsequent week following the test. For a period of two days preceding each field visit, the participants refrained from engaging in strenuous physical activities (i.e., they only engaged in low-load training sessions aimed at reinforcing standard game strategies and enhancing team cohesiveness) and avoided the consumption of supplements that might have ergogenic or synergistic effects, as indicated by previous studies [34]. Moreover, on the evening prior to each experimental session, the participants were provided with a standardized dinner (plain pasta (100 g), grilled chicken breast (180 g), and a typical medium-sized banana (~100 g)) rich in carbohydrates (approximately 65% of total energy intake). They arrived at the basketball court between 8:00 and 8:30 am following an overnight fasting period. To mitigate the influence of circadian rhythms, all experimental conditions were standardized to occur at the same time of day. The basketball court conditions were consistent across all experiments, with an approximate air temperature of 23–25 °C, barometric pressure of 1005–1025 mmHg, and the relative humidity maintained at approximately 45–50%.

Prior to commencing any experiment, the participants voided their bladders. Before engaging in any performance-based assessments, the participants completed a standardized 20 min warm-up routine. This warm-up regimen encompassed activities such as low-intensity jogging, dynamic whole-body stretches, and short intervals of high-intensity running. Moreover, prior to each test, all measurement instruments were calibrated in accordance with the manufacturer’s specifications.

All participants refrained from the use of any alcohol-containing beverages or medications, were in good general health, reported feeling well, resided at low altitudes (<1500 m), did not serve as blood donors throughout the experimental procedures, and reported no gastrointestinal discomfort post-supplementation. The participants were kept unaware of their performance, and they were discouraged from discussing the study with others to prevent the introduction of any expectations, whether positive or negative, until the project’s completion. This study was conducted during the early phase of the regular season in November, with the presumption that participants had not yet accumulated significant fatigue from an extensive series of matches. Moreover, there was a minimum interval of 4 days between the tests and the preceding matches to ensure adequate recovery and minimize the potential performance influence from recent competitive engagements.

### 2.4. Field Basketball Exercise Simulation Test (BEST)

The BEST involved a circuit-based activity lasting 30 s and encompassing various movement distances (standing/walking, jogging, running, sprinting, low shuffling, high shuffling, and jumping), as adequately described elsewhere [33]. The participants performed these circuits continuously for a duration of 12 min with the goal of completing a maximum of 24 circuits within the allocated time frame. During a full 12 min BEST trial, the participants covered a total distance of approximately 1725 m. This distance comprised three distinct activity components (Figure 2): low-intensity activity, accounting for 727 m (42% of the total distance), involving standing, walking, and jogging; high-intensity activity, covering 826 m (48% of the total distance), including running and sprinting; and shuffling activity, representing 172 m (10% of the total distance). In cases where the participants were unable to complete a circuit within the 30 s interval, no rest was provided, and they were immediately required to initiate the subsequent circuit. Consequently, in such instances, the participants did not achieve the targeted quantity of circuits (24) during testing unless they were able to restore the appropriate circuit timing.

The assessment of performance during the BEST encompassed the determination of the mean circuit and sprint times (CT and ST). The circuit and sprint times were meticulously recorded through the utilization of infrared photoelectric cells placed at a height of 1.1 m, which were seamlessly integrated into a timing system (Saint Wien Digital Timer Press H5K, Lu-Chou City, Taipei Hsien, Taiwan). This system exhibited a time resolution of 0.01 s and maintained precise measurement accuracy with an error margin of ±0.01 s. Additionally, HR data were acquired via telemetric monitoring (Polar RCX5, Polar Electro Oy, Kempele, Finland), with measurements collected at 5 s intervals throughout the duration of the BEST to facilitate the assessment of the average HR during the test.

The rationale for selecting the BEST as our assessment tool stems from its congruence with the distances reported in elite and sub-elite adult male basketball competitions [35]. In such contexts, the distribution of covered distances closely mirrors our chosen test, with low-intensity activity typically comprising approximately 40–44% of the total distance, high-intensity activity constituting approximately 47–51% of the total distance, and shuffling activity representing approximately 3–4% of the total distance. Furthermore, the BEST demonstrates strong indications of reliability and validity as a game-specific evaluation tool for the comprehensive assessment of both anaerobic and aerobic fitness related to basketball [33]. This is substantiated by its high test–retest reliability, which is characterized by high intra-class correlation coefficients ranging from 0.92 to 0.99 for mean sprint and circuit times [33].

### 2.5. Supplement Administration

For the purpose of this study, we employed two distinct supplementation regimens that were previously tested [22]. The experimental treatment, administered only to participants in the EXP group in the POST condition, comprised a combination of 84 mg of mangiferin (presented as 140 mg of Zynamite^®^, Nektium Pharma S.L., Las Palmas, Spain) and 140 mg of quercetin (provided as 280 mg of Sophora Japonica flower extract, Aki Organic, Repentigny, QC, Canada). In contrast, in the PRE and POST conditions, participants in the CON group received the placebo treatment, consisting solely of 420 mg of organic, gluten-free chickpea flour (Doves Farm Foods Ltd., Hungerford, UK). In the PRE condition, the EXP group received the control treatment. Both treatments were administered as a single dose precisely one hour prior to the BEST. The participants ingested the capsules along with 500 mL of water for hydration purposes. The experimental design followed a double-blind, cross-over, and counterbalanced approach, utilizing the Latin square method to ensure optimal randomization. To maintain blinding, both treatments were encapsulated in pullulan non-transparent capsules (Pullulan #1, LFA Machines Oxford Ltd., Bicester, UK), rendering them indistinguishable in taste, smell, and appearance. It was also expected that the conditioning levels of the participants, coupled with the relatively brief nature of the test trials, would mitigate the likelihood of training-related adaptations occurring due to repeated exposure during the study [36]. The selection of a singular dosage comprising 84 mg of mangiferin and 140 mg of quercetin as the experimental treatment was derived from a previous laboratory investigation [22]. That study demonstrated the potential of this supplementation regimen, administered one hour before exercise, to enhance repeated sprint performance [22].

### 2.6. Other Measurements

Blood samples were collected from the left index fingertip, yielding 7 μL of capillary blood, and subsequently subjected to an analysis of blood lactate (La^−^) levels (StatStrip® Xpress™ Lactate, Nova Biomedical, Waltham, MA, USA) [37]. Each blood sample was subjected to dual measurements, and the resulting values were averaged for the subsequent statistical analysis. The manufacturer’s internal studies reported coefficients of variation for typical imprecision, encompassing both within-run and day-to-day variability, which ranged from 3.4% to 5.9% for lactate values of 2.6 to 10.5 mmol·L^−1^ [38].

After performing three squat positions, each participant underwent a 3 s palpation, followed by the completion of a muscle soreness questionnaire. This questionnaire required participants to rate the perceived level of overall muscle soreness (RPMS) in the leg muscles, specifically the quadriceps, hamstrings, gastrocnemius, and tibialis, for both legs. Ratings were provided on a scale ranging from 0 (indicating the absence of soreness) to 10 (indicating a high level of soreness) [39].

Additionally, the RPE was quantified using the 6–20 Borg scale [32]. The subjective rating of perceived exertion served as an evaluative measure of overall muscular effort and the presence of fatigue. The blood lactate levels, RPMS, and RPE were assessed in a resting state 55 min prior to and 10 min following the BEST.

### 2.7. Statistical Analysis

Assumptions of the normality and homogeneity of variances were met by the data (Shapiro–Wilk *p* > 0.05, Levene’s test *p* > 0.05). For comparisons of subject variables and dependent variables between groups in the rest condition prior to the BEST, independent t-tests were applied. A 2 × 2 (grouped by condition) mixed analysis of variance (ANOVA) with repeated measures on the condition factor was performed to evaluate the influence of group (EXP and CON) and condition (PRE and POST) on the dependent variables (i.e., the mean CT, ST, and HR during the BEST and the La^−^, RPMS, and RPE after the BEST). For statistically significant interactions, Bonferroni post hoc pairwise comparisons were conducted. The analysis was performed using the SPSS software platform (version 29.0, IBM Corp., Armonk, NY, USA), and a significance level of α = 0.05 was chosen for all statistical analyses. Furthermore, a post hoc power analysis was conducted using GPower 3.1.9.2 software (Heinrich Heine University, Düsseldorf, Germany). The analysis employed the mean circuit time as the criterion variable, considering the following parameters: an effect size of d = 1.00, a significance level of (α) = 0.05, a sample size of 38, and a design comprising two independent groups that received experimental and placebo treatments. The resulting observed power (1 − β) exceeded 0.91.

## 3. Results

All results are presented as mean (M) ± standard deviation (SD) [95% confidence interval (CI)]. The descriptive characteristics (see Table 1) and pre-game measurements did not show statistical significance between the groups (*p* > 0.05). The dependent variables (i.e., the mean CT, ST, and HR during the BEST and the La^−^, RPMS, and RPE after the BEST) of both groups (EXP and CON) are presented in Table 2.

**Figure 3:**
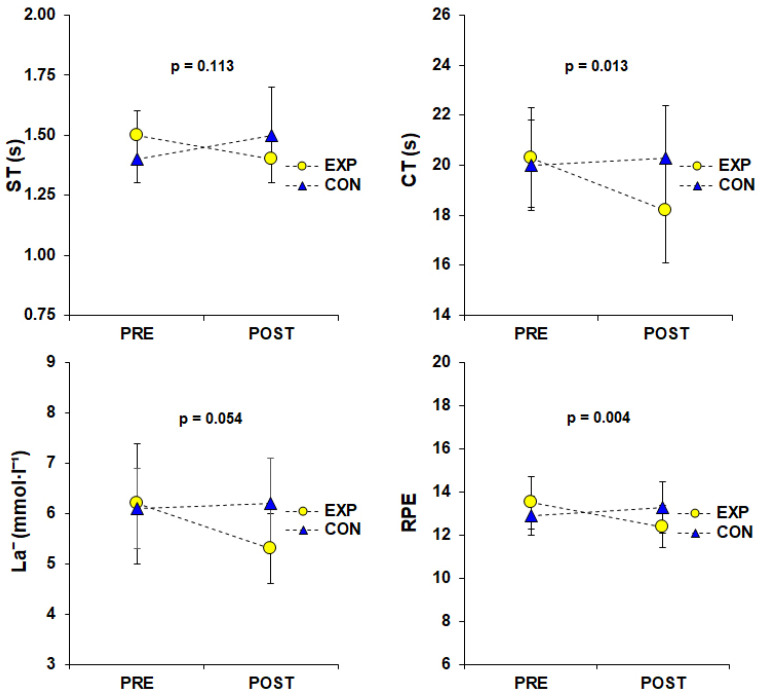
Interaction effects of mean ST and CT during the BEST and La^−^ and RPE after the BEST (condition vs. group). Error bars indicate standard deviations of the mean values. Abbreviations: BEST, basketball exercise simulation test; CT, circuit time; CON, control (group, Nc = 19); EXP, experimental (group, Ne = 19); La^−^, lactate; Nc, sample size of the control group; Ne, sample size of the experimental group; POST, condition where the CON group received a placebo supplement and the EXP group received an experimental supplement prior to the BEST; PRE, condition where both groups received a placebo supplement prior to the BEST; RPE, subjective rating of perceived exertion; and ST, sprint time.

The results of the mixed ANOVAs grouped by condition with repeated measures on the condition factor (Figure 3) indicated statistically significant interactions only for the mean CT values (F_1,36_ = 6.76, *p* = 0.013) and RPE values (F_1,36_ = 9.68, *p* = 0.004). The Bonferroni post hoc analyses of the significant interactions showed that for the mean CT and RPE values (Table 2), the EXP group had significantly lower values in the POST condition (18.17 ± 2.08 s and 12.42 ± 1.02, respectively) compared to the PRE condition (20.33 ± 1.96 s and 13.47 ± 1.22, respectively) and the POST condition of the CON group (20.31 ± 2.10 s and 13.32 ± 1.16, respectively) (*p* < 0.05). However, it is important to highlight the marginal non-significant interaction between the group and condition with respect to the mean La^−^ values (F_1,36_ = 3.96, *p* = 0.054), along with the notable main effect of the group (*p* = 0.029). The rest of the paired comparisons did not reach statistical significance. Additionally, Figure S1 illustrates the individual mean values of ST and CT during the BEST, along with the La^−^ and RPE values after the exercise (compared between conditions and groups).

## 4. Discussion

This study aimed to investigate the potential impact of mangiferin–quercetin supplementation on the performance of basketball players undertaking the BEST, a stop-and-go basketball-specific assessment. While our results demonstrate statistically significant improvements in the mean circuit time during the BEST and noteworthy reductions in RPE values following a singular dose of mangiferin–quercetin supplementation one hour prior to the test, we acknowledge the preliminary nature of these findings.

Aerobic exercise performance may be enhanced after the oral administration of quercetin [13]. A meta-analysis that examined the ergogenic potential of quercetin supplementation also showed that high doses (exceeding 600 mg) of quercetin administered over multiple days provide a statistically significant 3% improvement in endurance exercise capacity [11]. In contrast, another study found no discernible impact of quercetin supplementation (at a dosage of 1000 mg·day^−1^ for one week) on repeated sprint performance (involving 12 × 30 m maximal-effort sprints) in recreationally active, young adult men [40]. However, the efficacy of quercetin in the context of athletic performance remains a subject of uncertainty in some studies [13]. On the other hand, clinical trials involving MLE, notably abundant in mangiferin, have predominantly adopted single-dose study designs with a limited number of participants [20]. However, these trials provide initial clinical evidence indicating that the oral intake of MLE (e.g., 500 mg) has the potential to induce alterations in brain electrical activity, may enhance reaction time, and may mitigate fatigue and sensations of exhaustion [20]. Moreover, the combined administration of mangiferin and quercetin, when consumed within 48 h preceding a bout of repeated sprint exercise, demonstrates the capacity to mitigate the typical decrease in brain oxygenation observed during prolonged sprinting [18] and augment the extraction of oxygen by muscles [8,19]. Furthermore, a recent investigation illuminated the noteworthy ergogenic potential of combining MLE with quercetin, as evidenced by increased muscle power in fatigued female participants as well as improvements in peak $\dot{V}$O_2_ and brain oxygenation during prolonged sprinting [8]. In another relevant study, a singular dose comprising 84 mg of mangiferin and 140 mg of quercetin, administered one hour before a competitive event, followed by three additional doses at eight-hour intervals, exhibited potential in ameliorating muscle discomfort, diminishing damage, and expediting the recuperation of muscular performance [21]. Our results, consistent with the aforementioned studies, reveal an improvement in the mean circuit time during the BEST, along with a tendency for a decreased blood capillary La^−^ concentration, when participants were administered the mangiferin–quercetin supplement one hour before engaging in the BEST. Additionally, a noteworthy reduction in RPE values at the end of the test was observed.

Fatigue is an intricate phenomenon influenced by numerous factors governing the production and regulation of muscle contractions. Substances aimed at improving performance can exert their effects by enhancing energy availability and utilization; facilitating central command and motor control; and mitigating the negative consequences of energy depletion, reduced oxygen levels, and metabolite accumulation, as well as the impacts of reactive oxygen and nitrogen species (RONS) on force generation, muscle activation, and afferent sensory input. Stop-and-go sports such as basketball induce heightened glycolytic metabolism, leading to the accumulation of lactate and a decline in muscle pH [3,4,5]. Conversely, the process of acidification promotes the generation of hydroxyl radicals and diminishes the effectiveness of antioxidant enzymes [41]. Individual polyphenolic compounds such as mangiferin and quercetin possess distinct chemical characteristics that influence their specific interactions within various cellular compartments [42,43], and both polyphenols (mangiferin and quercetin) exhibit significant capabilities in neutralizing free radicals [10,14]. When utilized in combination, their cumulative antioxidant efficacy could potentially exceed that of the individual compounds [44,45]. The co-administration of quercetin and mangiferin may also be more effective in mitigating the RONS generated during exercise across different subcellular regions of skeletal muscle fibers compared to single compounds [46]. This could also happen by inhibiting the activity of xanthine oxidase and nicotinamide adenine dinucleotide phosphate-oxidase [47,48,49], which have a pivotal role as RONS sources during sprint exercise [50,51]. Moreover, RONS could influence calcium release and troponin sensitivity, affecting muscle power [15,16,52]. Interestingly, while a small dose of quercetin showed an ergogenic effect in our study, high doses of antioxidants might limit force generation [40,53]. This concept aligns with the idea that RONS impact muscle force according to an inverted U-shaped curve [54]. It is worth noting that longer-term supplementation with quercetin and vitamin C (125 or 250 mg·day^−1^) at different doses (500 to 1000 mg·day^−1^) did not significantly affect oxidative stress or antioxidant capacity [55]. Furthermore, cellular experiments have provided evidence that mangiferin enhances carbohydrate oxidation and mitigates lactate accumulation by enhancing pyruvate dehydrogenase activity [56,57]. In this current study, following the supplementation of mangiferin–quercetin, an improvement in the mean circuit time during the BEST was evident, while there was a noticeable trend toward a reduced blood capillary La^−^ concentration at the conclusion of the BEST.

The mean HR observed during the BEST in the EXP group remained remarkably consistent between the two conditions, indicating that the cardiac output did not undergo significant changes following mangiferin–quercetin supplementation. This consistency in heart rate suggests that skeletal muscle blood flow, and by extension, vascular conductance, which was presumed to be operating at maximal levels, likely remained unaltered [58,59]. Nonetheless, the co-administration of a singular dose of mangiferin and quercetin prior to repeated sprint exercise has demonstrated the capacity to augment muscle oxygen extraction [22]. This augmentation of oxygen extraction within the muscle tissue may enable an increase in muscle $\dot{V}$O_2_, subsequently offering the potential for a beneficial impact on BEST performance [60], such as improvements in mean circuit time. However, it is noteworthy that despite previous associations of the oral administration of 500 mg of MLE with improved reaction times and reduced fatigue [20] and the concurrent ingestion of a single dose of mangiferin–quercetin prior to three repeated Wingate tests demonstrating an enhancement of peak power output [22], the mean sprint time during the BEST did not exhibit a significant improvement in the EXP group.

Intense sprint exercise leads to the robust activation of type III and IV afferents, primarily due to the accumulation of metabolites such as lactate and hydrogen ions [61,62,63]. These sensory neurons are thought to play a significant role in the perception of effort and the sensation of exercise-induced pain. Mangiferin exhibits the capacity to cross the blood–brain barrier and exert influences on neurotransmission, potassium ion channels, and nociception processes [64]. This multifaceted impact may be attributed to its potential to reduce the stimulation of type III and IV muscle afferents, primarily mediated by RONS, and to modulate glycolytic activity and the accumulation of interstitial potassium ions [8]. Consequently, mangiferin could play a role in mitigating sensory feedback, providing a plausible explanation for the significant reduction in RPE values observed at the conclusion of the BEST following the administration of mangiferin–quercetin supplementation. However, it is worth noting that although the BEST protocol was not specifically tailored to induce muscle damage, as evidenced by the reported low levels of muscle soreness, the administration of a singular dose of mangiferin–quercetin did not appear to negatively impact the RPMS values upon completing the BEST. This observation aligns with the findings of a recent study [21].

Nonetheless, given the constraints of the current experimental design, our ability to provide a comprehensive explanation for this observed enhancement of performance (i.e., mean circuit time) is limited to offering preliminary insights. In contrast, our results offer an indirect indication of the potential ergogenic impact of the mangiferin–quercetin supplement during the BEST. Therefore, it is imperative that further investigations be undertaken to delve into the intricate physiological mechanisms underpinning this performance improvement.

### Strengths, Limitations, and Suggestions for Future Research

Despite the valuable insights gained from this study, several limitations should be acknowledged. First, the inclusion of exclusively male participants in our sample limits the generalizability of our findings. Second, this study employed a specific dose of mangiferin and quercetin in conjunction with a particular basketball test, which may not encompass the entire spectrum of potential dosages and performance assessments. Third, this study did not incorporate an evaluation of biochemical markers, which could have provided a deeper understanding of the physiological mechanisms at play. To enhance the applicability of our findings, further investigations in this field are warranted.

It is noteworthy that prior research has already demonstrated the performance-enhancing effects of combined mangiferin and quercetin supplementation among both male and female non-athletes [22]. Our study reinforces these previous findings. Furthermore, the compatibility of the distances covered during the BEST with those observed in adult male basketball competitions (e.g., low-intensity activities: 40–44%; high-intensity activities: 47–51%; and shuffling: 3–4% [2,3,5,35]) underscores the rationale for considering mangiferin–quercetin as a pre-game supplementation strategy for improving basketball performance. Nonetheless, to establish mangiferin–quercetin supplements as a new generation of natural plant-based food and supplement options within the sports industry, additional comprehensive, controlled clinical studies should be conducted across diverse athletic populations, utilizing a range of performance assessments.

## 5. Conclusions

The significance of our study lies in uncovering initial evidence suggesting that pre-game mangiferin–quercetin supplementation may positively influence sports characterized by intermittent high-intensity efforts, such as basketball. However, we recognize this study’s limitations in providing comprehensive evidence solely based on the effects observed in this controlled scenario. These findings prompt the need for more extensive investigations to validate and elucidate the mechanisms behind the observed performance enhancements in the broader context of stop-and-go sports.

## Figures and Tables

**Table 1:** Anthropometric and physiological traits (M ± SD [95% CI]) of EXP and CON groups.

Variables	EXP (Ne = 19)	CON (Nc = 19)
Height (cm)	200.43 ± 5.40 [198–202.86]	201.12 ± 4.71 [199–203.24]
Body mass (kg)	96.2 ± 7.61 [92.78–99.62]	97.67 ± 7.37 [94.36–100.98]
Body fat (%)	10.95 ± 1.82 [10.13–11.77]	11.11 ± 2.05 [10.19–12.03]
Age (year)	23.21 ± 3.08 [21.83–24.59]	24.05 ± 2.15 [23.08–25.02]
Experience practicing basketball (year)	12.53 ± 3.03 [11.17–13.89]	13.11 ± 2.47 [12–14.22]
Active in basketball competition (year)	11.21 ± 3.08 [9.83–12.59]	12.05 ± 2.15 [11.08–13.02]
Physical exercise training (h·week^−1^)	3.97 ± 1.11 [3.47–4.47]	3.79 ± 0.84 [3.41–4.17]
Basketball-related training (h·week^−1^)	10.63 ± 1.16 [10.11–11.15]	10.68 ± 1.2 [10.14–11.22]
$\dot{V}$O_2_max (mL·kg^−1^·min^−1^)	59.32 ± 2.65 [58.13–60.51]	59.05 ± 2.66 [57.85–60.25]
VT_2_ (%$\dot{V}$O_2_max)	78.26 ± 3.12 [76.86–79.66]	77.68 ± 2.98 [76.34–79.02]
HRmax (b·min^−1^)	196.21 ± 2.07 [195.28–197.14]	196.21 ± 1.72 [195.44–196.98]
CMJ height (cm)	47.11 ± 4.64 [45.02–49.2]	46.58 ± 4.21 [44.69–48.47]

Abbreviations: BF, body fat; BM: body mass; CMJ, countermovement jump; CON, control (group); EXP, experimental (group); HRmax, maximum heart rate; M, mean; Nc, sample size of the control group; Ne, sample size of the experimental group; SD, standard deviation; VT_2_, second ventilator threshold; $\dot{V}$O_2_max, maximum oxygen uptake; and [95% CI], 95% confidence interval.

**Table 2:** The M ± SD [95% CI] of the dependent variables (i.e., mean CT, ST, and HR during BEST and La^−^, RPMS, and RPE after BEST) of EXP and CON groups in two conditions (PRE and POST).

	EXP (Ne = 19)		CON (Nc = 19)	
Dependent Variable	PRE	POST	PRE	POST
ST (s)	1.47 ± 0.11	1.39 ± 0.14	1.43 ± 0.12	1.45 ± 0.18
	[1.42–1.52]	[1.31–1.46]	[1.38–1.48]	[1.38–1.53]
^†‡^ CT (s)	20.33 ± 1.96	18.17 ± 2.08	20.02 ± 1.80	20.31 ± 2.10
	[19.45–21.20]	[17.20–19.14]	[19.14–20.89]	[19.33–21.23]
HR (b·min^−1^)	175.81 ± 5.80	176.46 ± 8.57	176.21 ± 6.29	174.15 ± 6.53
	[173.00–178.63]	[172.92–180.01]	[173.40–179.03]	[170.60–177.69]
^†^ La^−^ (mmol·L^−1^)	6.16 ± 1.17	5.32 ± 0.66	6.07 ± 0.80	6.19 ± 0.94
	[5.69–6.63]	[4.95–5.70]	[5.60–6.53]	[5.82–6.57]
RPMS (Likert scale, 0–10)	3.84 ± 0.90	4.11 ± 0.81	4.00 ± 0.58	4.42 ± 1.17
	[3.49–4.19]	[3.64–4.57]	[3.65–4.35]	[3.95–4.89]
^‡^ RPE (Borg scale, 6–20)	13.47 ± 1.22	12.42 ± 1.02	12.95 ± 0.91	13.32 ± 1.16
	[12.97–13.97]	[11.91–12.93]	[12.45–13.45]	[12.81–13.82]

^†^ Significant main effect of group at *p* < 0.05. ^‡^ Significant interaction at *p* < 0.05. See text for significant pairwise comparisons. Abbreviations: BEST, basketball exercise simulation test; CT, circuit time; CON, control (group); EXP, experimental (group); HR, heart rate; La^−^, lactate; M, mean; Nc, sample size of the control group; Ne, sample size of the experimental group; POST, condition where the CON group received a placebo supplement and the EXP group received an experimental supplement prior to the BEST; PRE, condition where both groups received a placebo supplement prior to the BEST; RPE, subjective rating of perceived exertion; RPMS, subjective rating of muscle soreness; ST, sprint time; SD, standard deviation; and [95% CI], 95% confidence interval.

## Data Availability

The raw data supporting the conclusions of this article will be made available by the corresponding author upon reasonable request once all relevant substudies are reported and completed. The study protocol, data dictionary, and statistical analysis plan can also be made available by the corresponding author upon request. The data are not publicly available due to are part of an ongoing study.

## Supplementary Materials

The following supporting information can be downloaded at: https://www.mdpi.com/article/10.3390/nu16010170/s1, Figure S1: Individual values of mean ST and CT during, and La^−^ and RPE after the BEST (Condition vs. Group).
