# Supplementary figures and images for: Effects of a Singular Dose of Mangiferin–Quercetin Supplementation on Basketball Performance: A Double-Blind Crossover Study of High-Level Male Players
Source: Nutrients. 2024 Jan 4;16(1):170. doi: 10.3390/nu16010170 (PMC10781150; doi:10.3390/nu16010170)

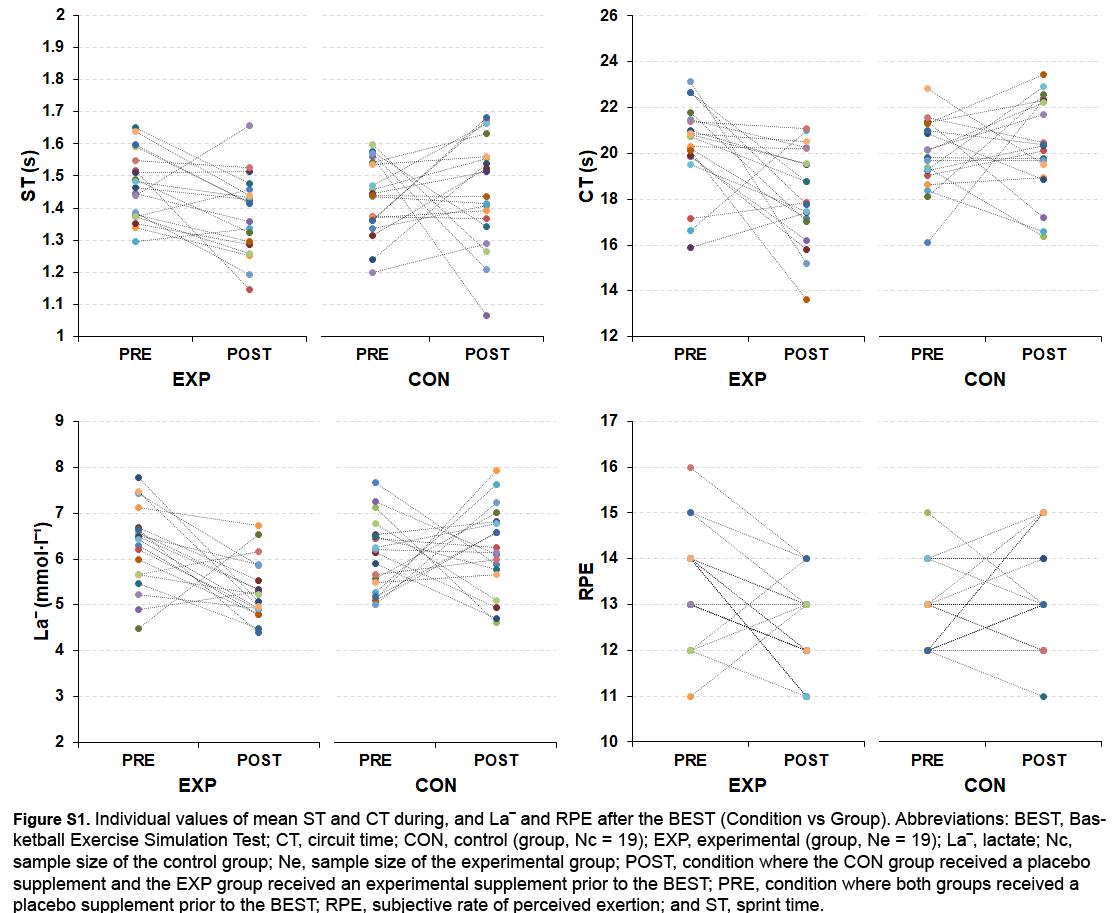

Supplement: Supplementary file 1 [file nutrients-16-00170-s001.zip › nutrients-2764516-supplementary.tif]
